# Categorization of Natural Dynamic Audiovisual Scenes

**DOI:** 10.1371/journal.pone.0095848

**Published:** 2014-05-01

**Authors:** Olli Rummukainen, Jenni Radun, Toni Virtanen, Ville Pulkki

**Affiliations:** 1 Department of Signal Processing and Acoustics, Aalto University, Espoo, Finland; 2 Institute of Behavioural Sciences, University of Helsinki, Helsinki, Finland; Centre Hospitalier Universitaire Vaudois and University of Lausanne, Switzerland

## Abstract

This work analyzed the perceptual attributes of natural dynamic audiovisual scenes. We presented thirty participants with 19 natural scenes in a similarity categorization task, followed by a semi-structured interview. The scenes were reproduced with an immersive audiovisual display. Natural scene perception has been studied mainly with unimodal settings, which have identified motion as one of the most salient attributes related to visual scenes, and sound intensity along with pitch trajectories related to auditory scenes. However, controlled laboratory experiments with natural multimodal stimuli are still scarce. Our results show that humans pay attention to similar perceptual attributes in natural scenes, and a two-dimensional perceptual map of the stimulus scenes and perceptual attributes was obtained in this work. The exploratory results show the amount of movement, perceived noisiness, and eventfulness of the scene to be the most important perceptual attributes in naturalistically reproduced real-world urban environments. We found the scene gist properties openness and expansion to remain as important factors in scenes with no salient auditory or visual events. We propose that the study of scene perception should move forward to understand better the processes behind multimodal scene processing in real-world environments. We publish our stimulus scenes as spherical video recordings and sound field recordings in a publicly available database.

## Introduction

A real-world scene can be considered as spatial and temporal context that can be found in real life, where events, including human action, can occur. The human brain is constantly selecting and combining information from different sensory channels to structure the external physical environment, but the amount of sensory stimulation is too large to be fully processed by our limited neural resources. A bottom-up process is thought to control the early stages of scene processing, where sensory information is selected for further analysis based on perceptual saliency [1]. In real-world scenes, however, top-down task-dependent information is also biasing the sensory selection process [2]. We live in a continuous world where we know where we are, where we came from, and what we are going to do next. We are also experts in perceiving our everyday surroundings, where the sensory information from different modalities integrates seamlessly together and corresponds to our expectations of the present situation. These conditions are hard to replicate in a controlled laboratory environment, and thus investigating real-world audiovisual scene processing in an ecologically valid setting is difficult. In the study at hand we strive for high ecological validity by employing surrounding visual projections along with spatial audio reproduction, and examine audiovisual scene processing in urban environments. The aim is to study the perceptual dimensionality of natural scene processing and to achieve a starting point for more detailed modeling of multimodal scene processing in real-world environments. By natural scene we refer to a reproduced real-world scene that could be encountered in our everyday lives, instead of artificial stimulation such as simplified visual shapes and pure tones.

The perception of real-world scenes has been studied by analyzing saliency maps, the gist in the visual domain, and the soundscape in the auditory domain. Unimodal saliency maps have been shown to accurately predict the focus of attention especially in the visual domain [3]–[5], but also auditory saliency can be modeled [6]. A saliency map decomposes stimuli into features that are most meaningful for a human perceiver. In addition to visual saliency models, visual scene gist can be modeled computationally [7]–[9]. Visual gist refers to a holistic mental representation of our surroundings, for example we are able to determine very rapidly whether we are in an open or closed space based on visual information, even before any impact from focused attention [10]–[13]. The auditory scene is analyzed in perceptual streams linked to sound events, where relevant sound information is integrated to a single stream according to its time-frequency domain properties [14]. In real-world environments, where many sound events occur simultaneously and in conjunction with events in other modalities, the auditory scene analysis based solely on auditory stimulus features becomes challenging, and the listener's goals and subjective reasoning become more influential in the stream formation process [15], [16].

Considering the perception of real-world environments, there is no clear knowledge about what environmental sounds people actually hear when they are not consciously listening for a particular sound [15]. The effect of attention and other sensory modalities, most importantly vision, on the auditory scene analysis is a little known area. Visual stimulation can bias the auditory perception [17], and vice versa [18], making the use of unimodal saliency modeling problematic. Recently, attention has been shown to have an effect on auditory streaming as well [16], [19], which would imply that the auditory scene can be structured differently based on the focus of attention. The study of urban soundscapes has revealed two generic cognitive categories: event sequences and amorphous sequences, referring respectively to soundscapes where individual sounds can or cannot be easily distinguished within the soundscape [20]. It appears that auditory stimuli are processed preferably as parts of a meaningful event in perceptual streams, or secondarily, in a more abstract manner along physical parameters if source identification fails. Recognizing the category of a complex soundscape (i.e. is the listener in a park or in a cafe) has been found to rely heavily on identification of the sound sources in the scene and inferring the category from the source information, while neglecting spatial information [21]. Sound has also a strong emotional impact on humans. The most prominent emotional dimensions of a soundscape experience have been identified as the perceived pleasantness and induced arousal [22], [23]. These are often related to the amount of natural versus mechanical sounds, natural sounds being perceived as pleasant and mechanical sounds as unpleasant and arousing.

Combining auditory and visual information seems to be beneficial for human perception in speech comprehension [24], spatial orienting efficiency [25], [26], and spatial localization accuracy [18]. Both covert and overt attention are directed differently when we encounter audiovisual stimuli in contrast to purely visual stimulation [25], [27], [28]. Computational modeling of audiovisual saliency is also attempted for restricted scenes, where sound has been found as a modulating factor for visual saliency [29], or audiovisual saliency has resulted from a linear combination of unimodal saliences [30]. From a wider viewpoint, evidence for bimodal effects on real-world scene perception have been presented in the field of environmental psychology, where tranquility has been shown to arise from a combination of naturalness in the visual world and the overall loudness and naturalness of the soundscape [31], [32].

Finally, the applicability of unimodal saliency maps to real-world audiovisual scenes seems questionable without taking into account the integration of sensory information and its effects on attentional control. Therefore, as already stated, the aim of this study is to further understand the perceptual dimensionality in audiovisual perception of natural scenes. This is done by evoking real-world scene experiences in a laboratory setting through ecologically valid audiovisual reproduction, and asking participants to categorize the scenes based on their perceived similarity. The empirical goal is to acquire a mapping of a diverse set of audiovisual stimuli along with perceptual attributes to study the human interpretation of real-world urban scenes. Our primary interest is to find whether a group of people is able to create a consistent low-dimensional perceptual representation for a set of natural scenes, and what are the perceptual attributes that are referred most often. Furthermore, objective environmental variables related to the loudness, dynamic visual information and indoor vs. outdoor classification are computed from the stimulus scenes, and fitted to the obtained perceptual mapping. Our hypothesis here is that the perceptual map originates from the physical world, instead of, for example, from functional attributes of the depicted spaces, and the subjective perceptual mapping should be predictable by physical properties of the stimuli.

A two-dimensional perceptual map of audiovisual scenes was derived in our study, confirming our hypothesis that a group of people describes natural scenes with similar perceptual attributes. The nature of our study was exploratory and thus we cannot precisely identify the relative contributions of the two sensory channels to the audiovisual scene perception. However, from the perceptual map we can draw evidence that the most important perceptual attributes in natural scenes depicting urban environments are the amount of movement, perceived noisiness, and eventfulness of the scene. Scene gist properties openness and expansion were found to remain as important attributes in scenes with no salient auditory or visual events.

## Materials and Methods

### Ethics statement

The participants voluntarily registered for the study through an online scheduling program in advance after reading an outline of the study and its aim. In addition, the participants provided verbal informed consent to voluntarily participate in this study at the examination day after receiving detailed information about the contents of the study, and they were aware that they could withdraw from the study at any time. It was also stated, that the gathered data was anonymous and not traceable back to the participants. Only demographic information about the participants was collected. There is a voice recording made of every participants' interview confirming the verbal consent. Therefore, no written informed consent was considered necessary, as outlined in the Finnish Advisory Board on Research Ethics' proposal [33]. In addition, the Ethics Review Board of Aalto University was consulted and an ethics review was found unnecessary. The research was conducted in the country of residence of the authors.

### Participants

A total of 30 naive participants took the test. They were recruited through social media and Aalto University's student mailing list. Nine of the participants were female and the average age was 26.5 (SD  = 6.6). All the participants reported to have normal or corrected-to-normal vision and normal hearing. No acuity screening or audiogram measurements were considered necessary, because the participants were supposed to perceive and assess the reproduced environments as they would experience ordinary situations in their everyday lives without relative weighting of the modalities. All the participants, except for one, were familiar with most of the locations of the outdoor scenes in the study, and reported to live or having lived in the Helsinki metropolitan area. Each participant reported having Finnish as their native language. The participants received a movie ticket as a compensation for their contribution. The authors did not participate in the experiment.

### Catalog of environments

**Figure 1:**
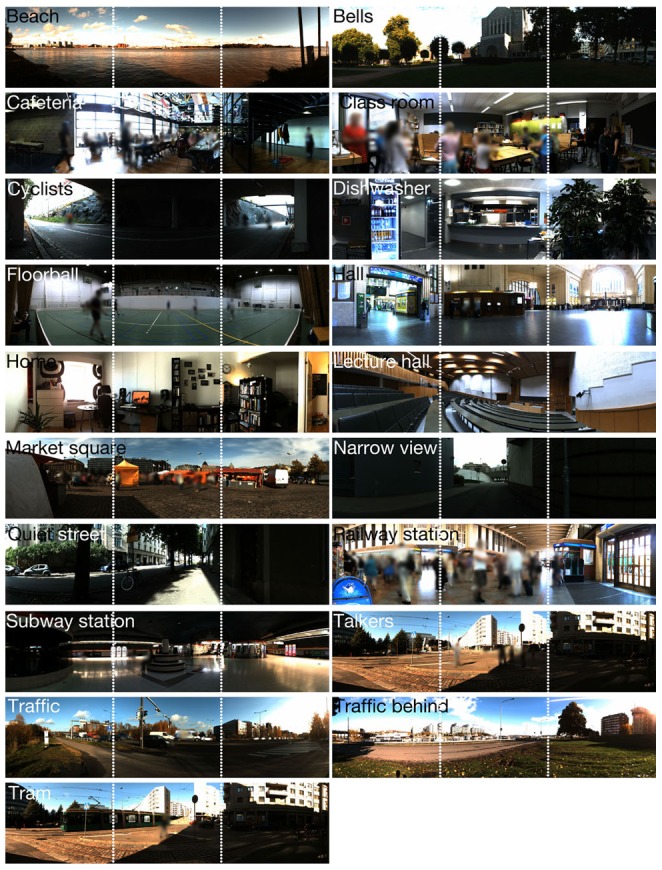
Collage of the visual contents. Dotted lines denote corners of the screens.

All stimulus scenes were recorded either indoor, or during summertime in an urban environment in the greater-Helsinki area, Finland. The aim was to provide as rich stimulus set as possible with clear perceptual (both auditory and visual) gradients, even though the requirement of finding a real-world environment with desired properties was sometimes challenging. The scene duration is 15 s, and the sequences are constructed so that the scene, and the events taking place in the scene, stay as invariant as possible. For example, in the scene #*Tram*, the passing-by event lasts for the whole duration of the scene. The recording device, and thus the observer, stays static in all the cases. The stimuli used in the experiment are listed in Table 1, along with an *a priori* categorization of the scenes according to their objective environmental attributes. A frame capture of each content is shown in Figure 1. In addition, the stimulus scenes are available for preview and for full-resolution download at: http://www.acoustics.hut.fi/go/plosone14-avscenes/ (username: *avscenes*, password: *isotVIDEOT*). The scenes #*Cafeteria* and #*Class room* are not published due to limited rights of publication.

**Table 1:** Catalog of the scenes and a description of the content.

Short name	Setting	Atmosphere	Soundscape	Description
Beach	Outdoor	Calm	Quiet, 41 dB	Expansive view of the sea and quiet
Bells	Outdoor	Calm	Quiet, 52 dB	Calm park with church bells ringing
Cafeteria	Indoor	Busy	Noisy, 64 dB	Busy elementary school cafeteria
Class room	Indoor	Busy	Noisy, 63 dB	Busy elementary school class room
Cyclists	Outdoor	Calm	Quiet, 48 dB	Restricted view of a downtown cycle path
Dishwasher	Indoor	Calm	Noisy, 63 dB	Close up view of a dishwasher
Floorball	Indoor	Busy	Quiet, 59 dB	Indoor hall with a game of floorball
Hall	Indoor	Calm	Quiet, 54 dB	Large departure hall with a few people
Home	Indoor	Calm	Quiet, 41 dB	Small room with TV on
Lecture hall	Indoor	Calm	Quiet, 38 dB	Large and empty lecture hall
Market square	Outdoor	Busy	Quiet, 58 dB	Market square with a few stalls and people
Narrow space	Outdoor	Calm	Quiet, 53 dB	Space limited by walls but expansive view
Quiet street	Outdoor	Calm	Quiet, 43 dB	Quiet street with overhead trees
Railway station	Indoor	Busy	Noisy, 62 dB	Large and busy departure hall
Subway station	Indoor	Calm	Noisy, 58 dB	Large and calm space with a few people
Talkers	Outdoor	Calm	Quiet, 54 dB	Few people talking at a street corner
Traffic	Outdoor	Busy	Noisy, 71 dB	Busy street in front of the viewer
Traffic behind	Outdoor	Calm	Noisy, 59 dB	Busy street behind the viewer
Tram	Outdoor	Busy	Noisy, 74 dB	Loud tram passing close by

The stimulus selection was based on previous studies of categorization of environmental scenes, where the scenes were divided according to their superordinate, basic and subordinate levels of categorization [34]. Each of the scenes in this study can be viewed as depicting a basic level scene, such as home or school, with the dynamic features of the scene determining the subordinate category. International Telecommunication Union's (ITU) recommendation defining the video content categories for subjective quality evaluation [35] was used as a guideline when choosing the temporal properties for the scenes.

### Reproduction

The experiment was conducted in an immersive audiovisual environment located at the Department of Signal Processing and Acoustics in Aalto University School of Electrical Engineering. The environment is built inside of an acoustically treated room and consists of three high-definition video projectors producing a horizontal field-of-view of 226° at the viewing position on three acoustically nearly transparent screens. The screens are 2.5×1.88 m each and installed to follow the shape of the base of a pentagon. The display area extends to the ground. Distance from the observation position to the center of each screen is 1.72 m. More detailed information about the technical specifications can be found in [36].

The videos were captured with a recording device capable of producing a spherical video (Point Grey Research: Ladybug 3). The videos were cropped to reproduce a 226° slice of the full circle on the screen, making the visual scene consistent with the auditory scene. The video was recorded and reproduced at 16 frames-per-second and the resolution of the final video was 4320×1080 pixels produced by the three projectors. With this resolution and viewing distance, the inter-line distance is 3.5 arcmin, which affects the sharpness of the image, but the objects in the scene are still easily recognizable.

**Figure 2:**
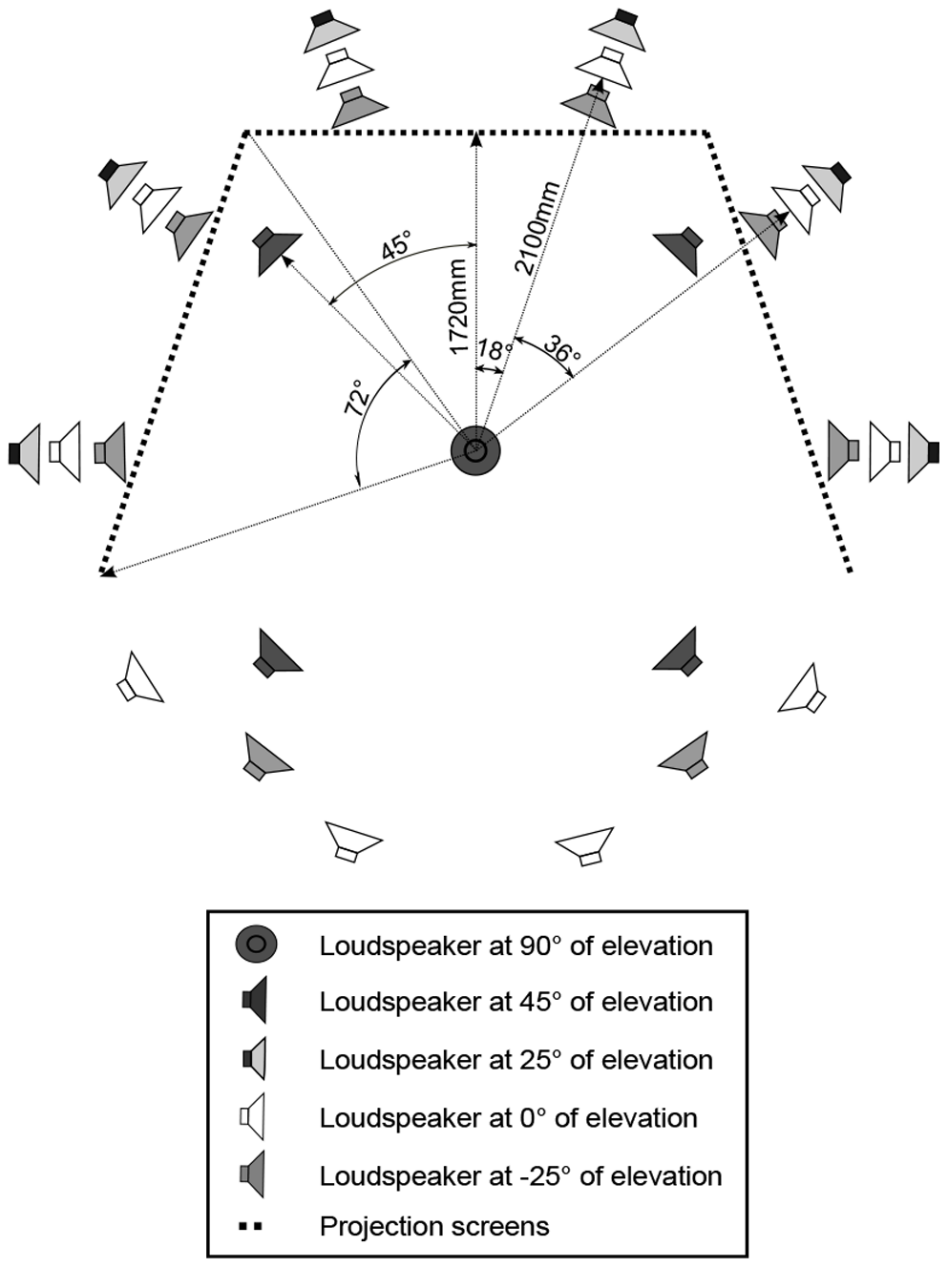
Loudspeaker setup and the projection screens. The observer is seated in the center. Adopted from: [36].

The audio reproduction system consisted of 29 loudspeakers (Genelec 1029). The loudspeakers were located on a sphere with a 2.1 m radius centered at the observation position. The loudspeaker layout and the projection screen setup are depicted in Figure 2. The signals to the loudspeakers were derived with Directional Audio Coding (DirAC; [37], [38]), which is a recently proposed parametric spatial audio technique. DirAC analyzes and synthesizes the sound field from A-format microphone (Soundfield SPS200) signals recorded from a live situation. The A-format microphone captures the sound field through four near-to-coincident cardioid capsules. DirAC was chosen to be used in the reproduction of spatial sound over the 29-loudspeaker layout, since in subjective testing with comparable layouts it has been found to provide a perception of sound prominently closer to the original perception than the reproduction provided with time-domain techniques that use the same input [39]. The A-weighted sound pressure level of each stimulus scene was set to match the one at the corresponding recording site.

### Procedure

First, the participants were familiarized with the stimulus scenes by watching and listening the whole list of 19 scenes presented in a random order. Thereafter, the participants were instructed to freely categorize the scenes into three or more groups, and to avoid groups with only single stimulus. The motives for the categories were left for the participants to decide. The categorization had to be based on the perceived similarity of attributes of the reproduced scenes. Examples of bad attributes were said to be forming groups based on personal familiarity with the depicted space or personal significance that the space might have for the participant. In addition, an analogous case of finding discriminating attributes between three orange juices was described to the participants. In such a case, the question is how to describe the juices and maybe recommend one of them to someone who has not tasted them. As one cannot discriminate between the juices by saying that they taste like orange, they should find something essential about the taste. Similarly with the scenes, it is maybe possible to describe a given scene in a way that sets it apart from some and resembles others.

**Figure 3:**
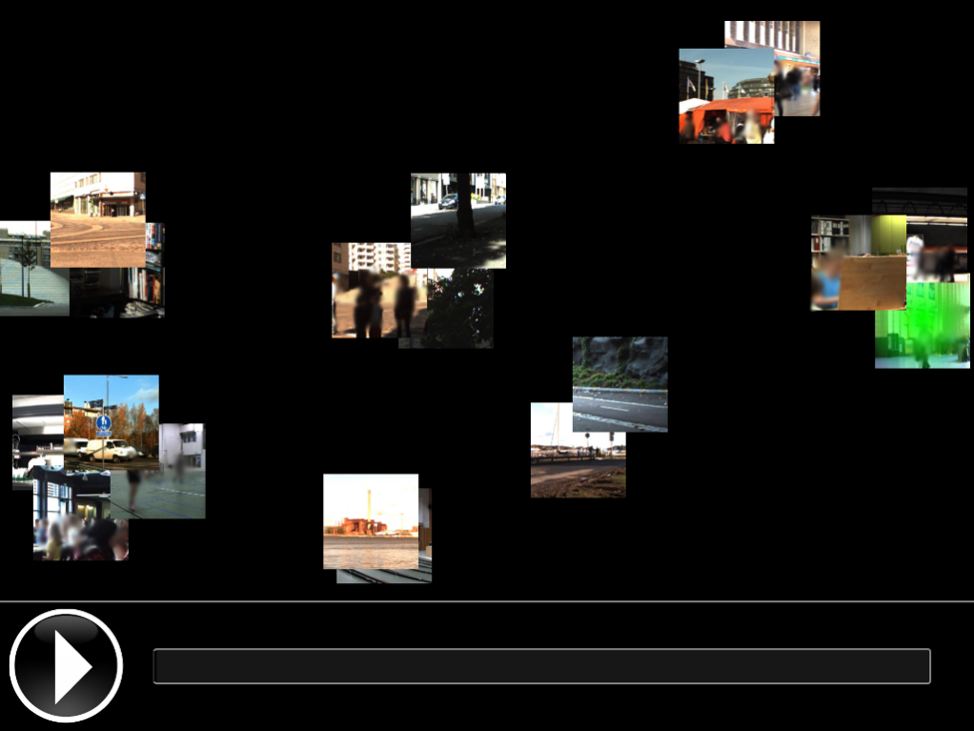
Graphical user interface on touch screen.

Participants performed the free categorization task on a tablet computer with a touch screen (Apple iPad2). The graphical user interface is depicted in Figure 3. The stimuli were illustrated on the screen by 100×100 px icons cropped from the video contents. Initially, the icons were randomly distributed on the touch screen and their purpose was to provide a mnemonic about the stimulus content but not to guide the grouping process. In addition to the icons, there was a *play* button that the participants could use to watch the stimuli as many times as necessary. Also, a progress bar was provided. A stimulus was selected for playback by touching the corresponding icon and clicking the play button. The stimulus would then be played once.

It was stressed to the participants that they were not required to determine a global categorization rule. They should not think of the screen as having x- and y-axis describing some global features, but rather, the screen provided them with a plane on which to build the categories, each with their own similarity attributes. After the participant was content with the acquired categorization, a semi-structured interview took place. All interviews were conducted by the same person (first author). In the interview, the participants were asked to give short descriptive names for the categories, and thereafter, each of the categories was examined by a two-level interview process. For each group, the interviewer first asked why the participant had allocated the chosen stimuli into the given category. The reasons were supposed to be related to the perceived attributes of the scene. Next, the interviewer asked more specifically about the properties of the attributes the participant had mentioned. Basically, the interviewer aimed at finding the underlying reasons affecting the categorization by asking consecutive “why”-questions about the attributes the participant had paid attention to. The interview was conducted in Finnish and the interviewer took notes of the answers. In addition, a voice recording was made of every interview in case the interviewer needed to verify details afterwards. On average, the entire test took 45 minutes to complete (*min*  =  30 min, *max*  =  80 min, SD  =  12 min).

### Statistical analysis methods

**Figure 4:**
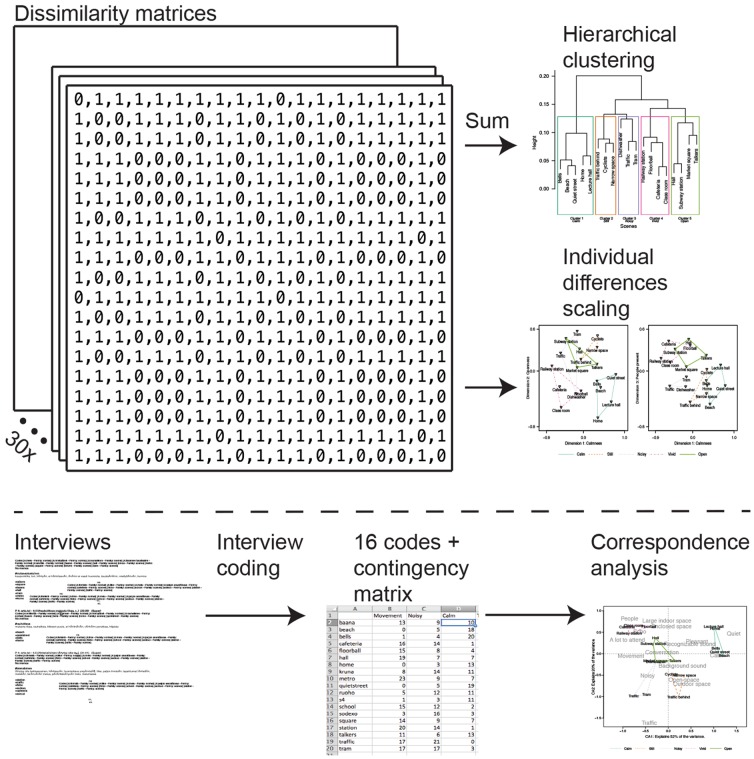
Schematic of the analysis methods and the data flow.

We used three different multivariate data analysis methods to inspect different aspects of our datasets, namely hierarchical clustering, multidimensional scaling (MDS), and correspondence analysis (CA). The analysis methods make different assumptions of the underlying data, the hierarchical clustering having the least amount of assumptions, and thus it can be used to verify the following two analyses. The MDS and CA methods use different datasets, that is, the dissimilarity matrices and the interviews. The MDS was necessary to inspect the variation in the participants' categorization strategies, and the CA was important in revealing the perceptual attributes' relation to the stimulus scenes. Finally, having similar overall ordination from the two methods, originating from different datasets, adds more support to the validity of both the solutions. Overall schematic of the analysis methods and the data used in each test is depicted in Figure 4. The different analysis methods and data preprocessing are introduced in the following.

#### Dissimilarity matrix

The perceived similarity of the scenes was extracted from the individually formed categorizations of the scenes. A symmetric and non-negative dissimilarity matrix with zero diagonal was formed for each participant. The matrix was constructed from pair-wise dissimilarities of the scenes by denoting scenes that were in the same category as having pair-wise dissimilarity value of 0 with each other, and dissimilarity value of 1 with scenes in other categories. An example of such dissimilarity matrix is depicted in Figure 4.

#### Hierarchical clustering

The idea behind hierarchical clustering is to obtain a baseline interpretation of the high-dimensional dataset. In further analysis, the baseline clustering can be used to validate the more detailed and subjective interpretations by inspecting if the clusters remain separated. In addition, by clustering the stimulus scenes into groups, the interpretation of perceptual maps in subsequent analysis is potentially easier. Overall dissimilarities were calculated by summing the 30 dissimilarity matrices together, and by using Bray-Curtis dissimilarity [40] to obtain a non-metric dissimilarity value for the scenes from the number of co-occurrences of the same scenes in the same category. Hierarchical clustering is initiated by combining the two most similar scenes (i.e. having the lowest dissimilarity score) into one cluster. Thereafter, the next fusion depends on the chosen clustering algorithm; in this study, the average linkage clustering [41] was used. In average linkage clustering, the fusions occur between group center points, instead of the nearest or the furthest neighbors as in single linkage or complete linkage approaches. Average linkage approach should ideally produce compact clusters and provide a compromise between single linkage and complete linkage algorithms, which tend to produce chain-like long clusters or be sensitive to outliers, respectively. Finally, the clustering result can be inspected at any level of fusion by cutting the clustering tree (dendrogram) at the desired height.

#### Multidimensional scaling

The dissimilarity matrices were further analyzed by multidimensional scaling (MDS), which is an exploratory data analysis technique well suited for identifying unrecognized dimensions that the participants could have used when making the categorization [41]. Essentially, MDS transforms subjective scene similarity judgments into distances in a multidimensional perceptual space. The dimensionality of the perceptual space has to be predefined, and typically solutions with different numbers of dimensions are tested to see which produces the smallest amount of stress between the original similarity judgments of the scene pairs and the Euclidean distances in the low-dimensional perceptual map. The MDS algorithm starts by randomly assigning the 19 scenes for example to a two-dimensional space. Next, the goodness-of-fit of the solution is evaluated by comparing the rank-order of the pair-wise Euclidean distances between scenes in the solution to the rank-order of the original similarity judgments. If the rank-orders do not agree, the 19 scene points are moved in the perceptual space to make the distance-based rank-order agree better with the similarity judgments. This process is repeated until a satisfactory fit is achieved between the distances and the similarity judgments. The algorithm is then run again with another dimensionality to see if a higher-dimensional space would produce better goodness-of-fit.

The individual dissimilarity matrices were analyzed using the R [42] implementation of the individual differences scaling (INDSCAL) algorithm using iterative stress majorization [43], which is a variant of MDS revealing also how similarly the participants were thinking about the dimensions present in the set of stimulus scenes. INDSCAL assumes that all participants share a common perceptual space, but have different individual weightings for the common space dimensions. The individual weights can be plotted in the formed common space, and individual differences analyzed by observing the distances from the axis. Participants positioned close to each other show similar weighting for the common perceptual dimensions. Furthermore, the participant's distance from the origin represents goodness-of-fit. Positions close to the origin have little weight on the common space and are considered to fit poorly to the proposed common solution.

#### Interview coding

**Figure 5:**
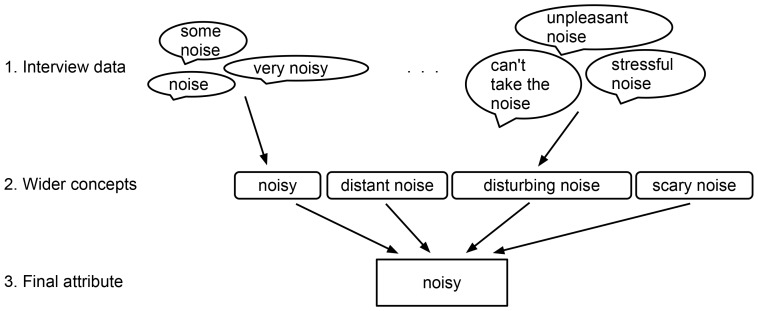
Coding process of the interview data.

The interview data was searched for expressions that could be coded under broader terms. The coding process was based on the grounded theory principle, where a phenomenon is analyzed and a theory formed beginning from the data [44]. The coding process introduces a possible error caused by the researcher's interpretation of the interview data. Therefore, the coding process was repeated by an outside researcher on five randomly selected interviews. The re-coding process was based on the interview notes taken by the interviewer. The qualitative analysis was done in Atlas.ti [45], where, in the first step, 355 unique codes were developed based on the data, and attached to the interview notes. The codes were related to the participants' descriptions of the perceptual attributes of the scenes (i.e., the reasons why certain categories were formed and scenes placed into them). Next, the number of codes was reduced by combining codes with similar meaning together under wider concepts. Figure 5 displays a diagram of how the coding process evolved. The interviews were conducted in Finnish, and the coding was also done in Finnish. As the final step, the obtained codes were translated into English preserving the meaning of the original code.

#### Correspondence analysis

Correspondence analysis (CA; [41]) was performed on the acquired interview data using the R-package *vegan* [46]. In CA, the characterization of meaning is based on verbal ratings of the scenes. The perceptual attributes, elicited through an interview process, are presented in a low-dimensional joint space with the stimulus scenes. For the CA algorithm, a contingency matrix was formed, where the rows corresponded to the 19 scenes, and the columns corresponded to the 16 most frequent interview codes. Next, the number of occurrences of each code in each scene were counted and marked into the matrix. The CA algorithm calculates a similarity measure for the codes in relation to the scenes, quite similarly as MDS used the similarity between scenes, and uses those similarities as the basis for the perceptual map. The strength of the CA is that it is able to display both the scenes and the codes in a joint perceptual space making the interpretation of the results easier.

The CA is able to attenuate the effect of categories formed based on higher-level cognitive reasoning, for example personal significance of a place or knowledge of the social function of the space. This kind of categories would be attached with codes with small number of references, and thereafter they would be ruled out of the actual analysis process, since only the most frequent codes were allowed into the CA model. In effect, the CA should reveal the underlying perceptual dimensions that actually stem from the perceivable physical environment.

## Results

### Subjective categorization of the scenes

**Figure 6:**
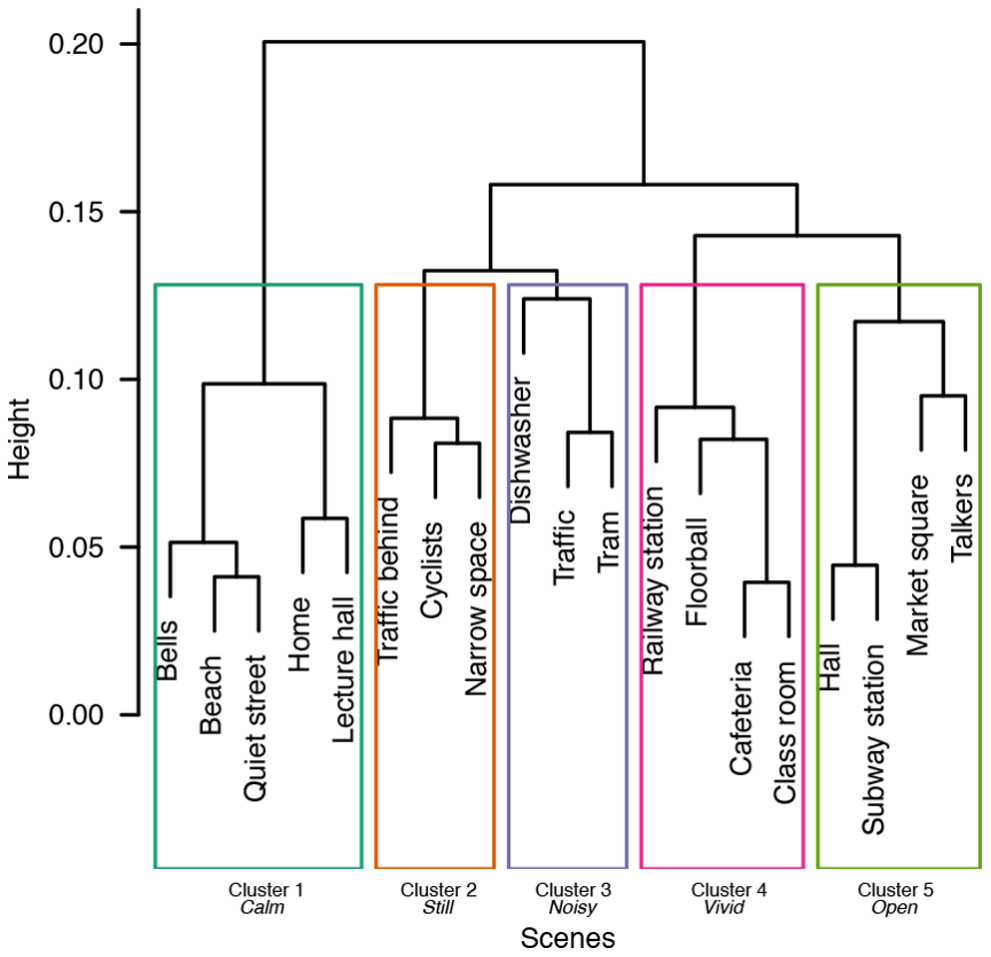
Dendrogram showing the hierarchical clustering result from average linkage clustering. The cluster tree is cut at level with five categories, and the resulting clusters are denoted by the rectangles. The clusters are holistically named based on the prevalent ambience of the given scenes.

Inspecting the subjective categorizations reveals that the participants formed 5.4 categories on average, with minimum amount of three categories and maximum of eight categories. Figure 6 displays the dendrogram resulting from the average-linkage clustering algorithm. The cluster tree is cut at level with five clusters, reflecting the participants' typical amount of formed categories (mode  = 5). The resulting clusters are highlighted with rectangles. In addition, holistic names describing the prevalent ambience stemming from the scenes in a cluster are presented.

Cluster #1 is the largest and contains calm sonic and visual environments, both from indoor and outdoor. Therefore, the holistic name given to Cluster #1 is *Calm*. Cluster #2 includes visually calm outdoor scenes with some ambient sound, well described by the word *Still*. In contrast, Cluster #3 contains noisy outdoor and indoor scenes with modest movement, therefore it is denoted *Noisy*. Cluster #4 consists of large indoor spaces with lots of people, movement and sound, in other words, the cluster is *Vivid*. Finally, Cluster #5 contains both outdoor scenes and large indoor spaces with people, modest movement, and some sonic events. The last cluster is best described as being visually *Open*.

### Dimensionality of the categorization and participant integrity

**Figure 7:**
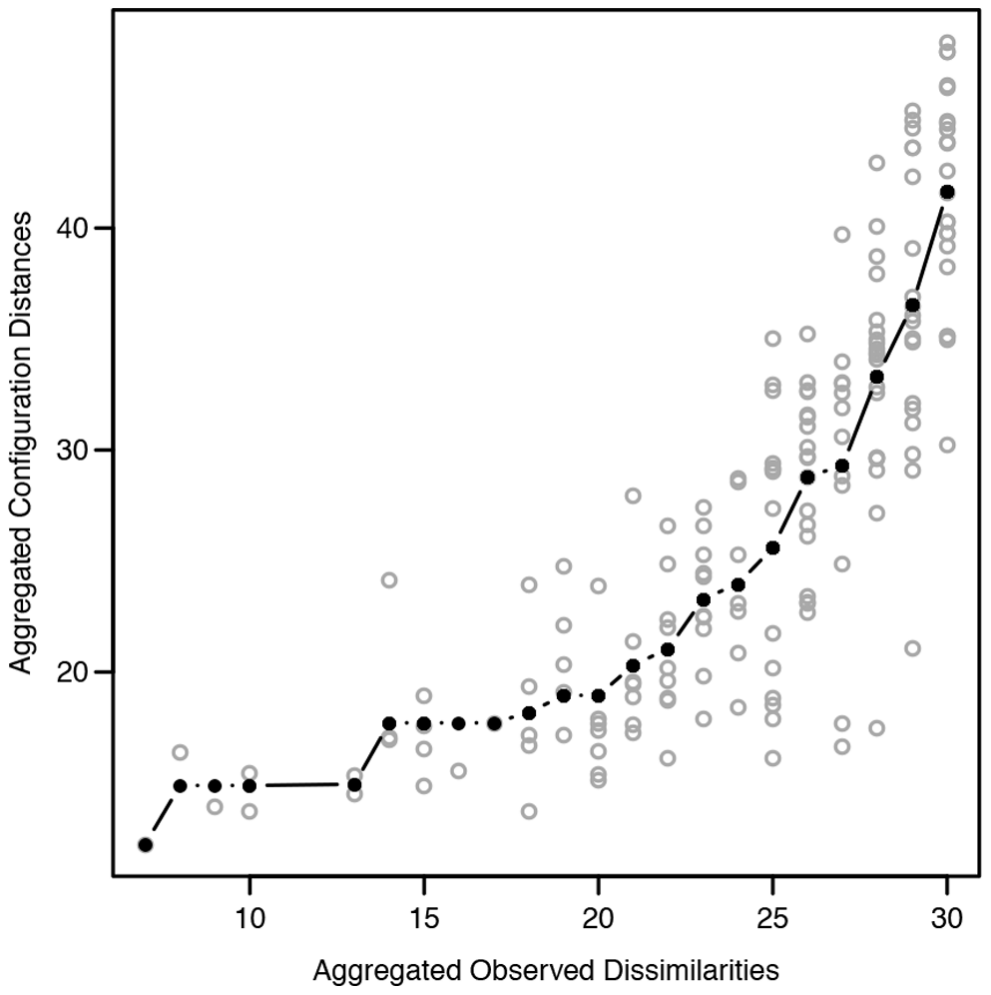
Shepard diagram displaying the observed dissimilarities against the fitted distances. The diagram is drawn for a three-dimensional individual differences scaling model. The black line denotes isotonic regression.

**Figure 8:**
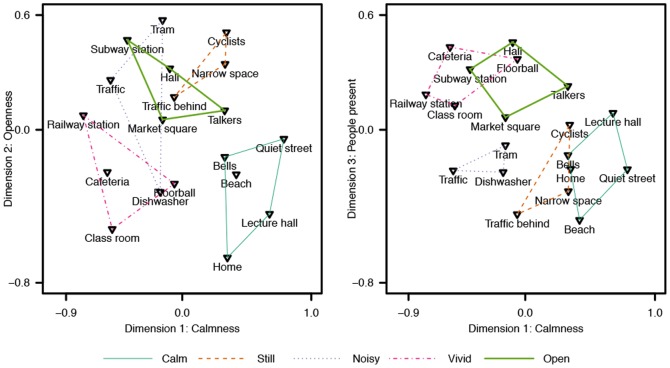
Perceptual map resulting from nonmetric multidimensional scaling with three-dimensional solution. Hierarchical clusterings are drawn on top of the ordination. Dimensions 1 & 2 are plotted on the left, and Dimensions 1 & 3 on the right. The dimension names are inferred from the weightings of the stimuli on the common space dimensions.

**Figure 9:**
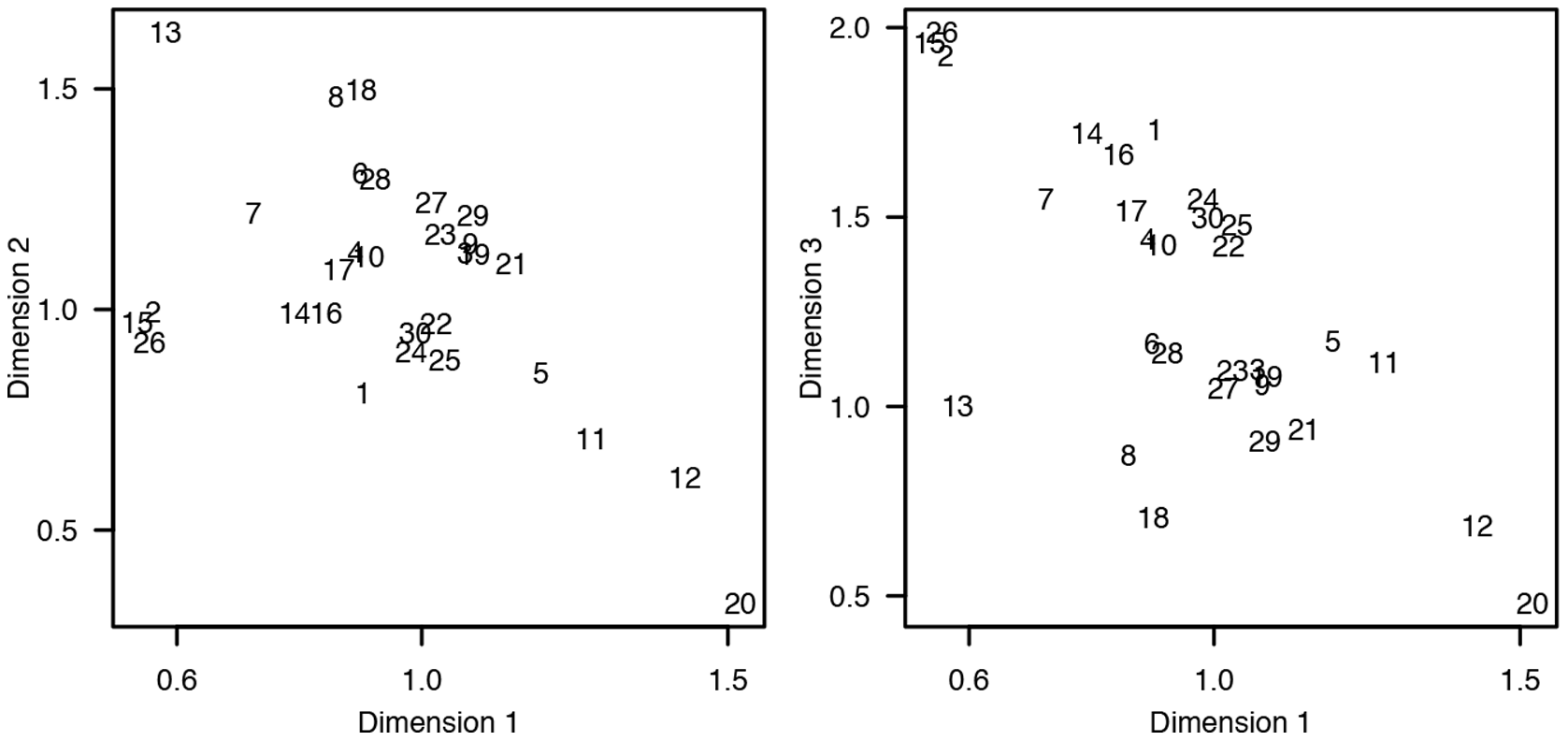
Participants' weights in the three-dimensional common space. Dimensions 1 & 2, obtained from the individual differences scaling analysis, are plotted on the left, and Dimensions 1 & 3 on the right.

Individual differences scaling (INDSCAL) models with dimensionalities ranging from two to five were fitted to the data, and the resulting nonmetric stress values were: .32, .16, .09, and .05, respectively. In this study, an INDSCAL model with three dimensions was found to explain the variance of the data with good quality (nonmetric stress  =  .16; [47]), and adding a fourth dimension did not provide easily interpretable information. Therefore, a model with three dimensions was chosen as suitable representation of the present data. Given the non-metric dissimilarity data, the mapping from the dissimilarities to distances in the perceptual map is non-linear. The mapping function is shown in Figure 7 which displays the Shepard diagram for the three-dimensional INDSCAL model. The isotonic regression curve in the Shepard diagram does not contain large steps indicating that the chosen model properly maps the non-metric dissimilarities to the three-dimensional common space. The grey circles in the plot show how a judged dissimilarity is mapped to the perceptual space. From the plot we can see that small dissimilarity values correspond well to small distances, and large dissimilarities correspond to large distances in the perceptual map. However, the mapping curve is clearly non-linear stating that absolute distances in the visualization are not accurate, and only relative distances should be inspected. Figure 8 presents the common space perceptual map in two-dimensions, first plot shows Dimensions 1 & 2 and the second plot Dimensions 1 & 3. In addition, the hierarchical clustering results are displayed on top of the ordination. The corresponding maps for individual weights in the common space are displayed in Figure 9 for the thirty participants.

Multidimensional scaling was performed on the categories formed by the participants without any interpretation. Therefore, categories based on higher-level cognitive functions, with little or no correspondence to the perceptual attributes of the space, affect the obtained result. However, it is beneficial to inspect the raw categorization and the participant integrity before interpreting the results further. In Figure 8, quiet and still scenes both outdoor and indoor receive the highest weights for Dimension 1, for example #*Quiet street* (Dimension 1  =  0.79) and #*Lecture hall* (Dimension 1  =  0.68). In contrast, noisy and busy outdoor and indoor scenes, for example #*Railway station* (Dimension 1  =  −0.77) and #*Traffic* (Dimension 1  =  −0.56), are negatively weighted for the Dimension 1. It can be stated that Dimension 1 appears to be related to the perceived calmness of the scenes, where both auditory and visual content affect the perception. The result is validated by observing Cluster #1 (*Calm*) having the highest weights and Clusters #3 (*Noisy*) and #4 (*Vivid*) having negative weights on Dimension 1.

Dimension 2, on the other hand, appears to yield the highest weights for outdoor scenes, or distinctly large indoor spaces, such as #*Hall* (Dimension 2  =  0.32) and #*Subway station* (Dimension 2  =  0.47). Indoor scenes and limited outdoor scenes receive negative weights on Dimension 2, therefore, it can be assumed to describe the openness of the scene. Temporal aspects, both auditory and visual, seem to have no impact on Dimension 2, since Clusters #1 and #4 containing the calmest and busiest scenes obtain similar weights. Finally, Dimension 3 is more difficult to characterize by examining the environmental attributes. It seems, that scenes with people present in the view, such as #*Hall* (Dimension 3  =  0.46) and #*Cafeteria* (Dimension 3  =  0.43), have the highest weights on Dimension 3, whereas scenes with no people have negative weights.

Inspection of Figure 9 for the individual weights of the participants in the common perceptual space reveals that no distinctive groups can be found. An important observation is that no participants are distinctively close to the origin that would indicate a poor fit to the common space dimensions. Instead, the participants are nicely grouped together especially in Dimensions 1 & 2. Participants #12 (Dimension 1  =  1.43) and #20 (Dimension 1  =  1.52) seem to have weighted the first Dimension more than the other two dimensions, whereas participants #2 (Dimension 3  =  1.93), #15 (Dimension 3  =  1.96) and #26 (Dimension 3  =  1.99) favor the third Dimension. Participant #13 (Dimension 2  =  1.63) seems to be inclined towards the second Dimension. The few individuals who have weighted a given Dimension more than the majority indicate that they may have understood the task differently, or used one global rule in forming the categories, whereas others have used a more diverse approach. Otherwise, having one large group of participants with no division to subgroups implies that there were no varying strategies to perform the categorization. However, a division to two subgroups can be observed in Dimensions 1 & 3, where some have favored the third Dimension slightly more than the others. This could imply that the presence or absence of people in the scene was more important to some.

### Categorization process

The individual motives for categorization were inspected by analyzing the interview data. Correspondence analysis was used to relate the elicited perceptual attributes to the stimulus scenes and, at this point, attributes with only a few references in the data were excluded. In the analysis, only the 16 most frequent codes, presented in Table 2, were used. Codes beyond that had less references, and contained negations (i.e., they were based on the absence of some attribute), which differs from the more frequent codes. The inter-rater agreement was found acceptable with Cohen's *κ*  =  .62 according to [48], who have set threshold values defining .60 ≤ *κ* ≤ .79 as substantial and *κ* > .80 as outstanding.

**Table 2:** Frequency of the codes in the interview data, and the suggested dominating modality.

Code	Frequency	Dominant modality
Movement	198	Visual
Noisy	188	Auditory
Calm	161	Audiovisual
Recognizable sound	138	Auditory
Background sound	130	Auditory
People	117	Audiovisual
A lot to attend	100	Audiovisual
Enclosed space	94	Visual
Quiet	92	Auditory
Conversation	78	Auditory
Large indoor space	76	Visual
Traffic	74	Audiovisual
Open space	73	Visual
Outdoor space	73	Visual
Pleasant	73	Audiovisual
Echo	66	Auditory

**Figure 10:**
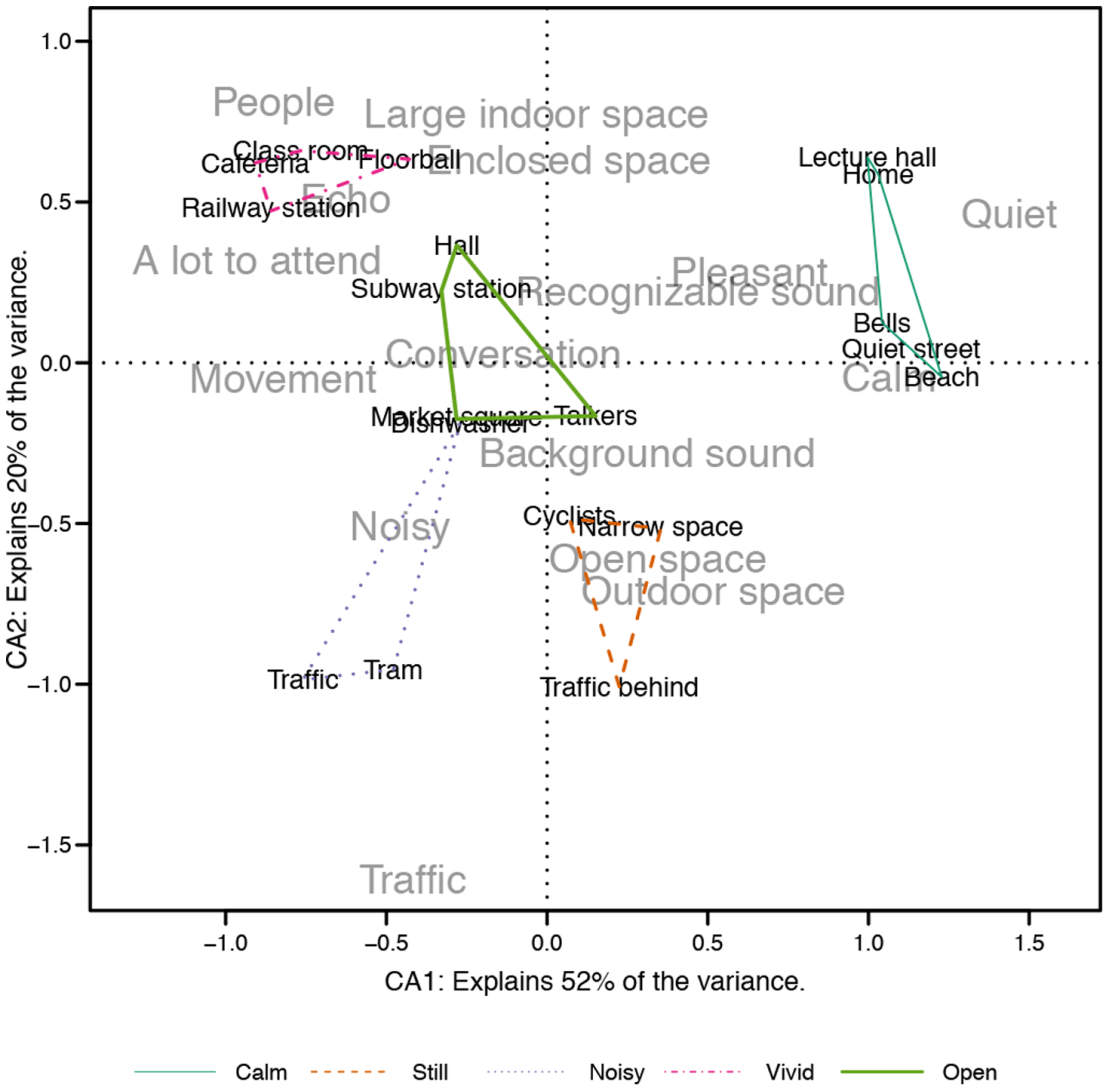
Correspondence analysis for the interview codings in Dimensions 1 & 2. Black denotes the stimulus scenes and grey denotes the codes. Hierarchical clusterings are drawn on top of the ordination.

From the 16 most frequent codes, five are related to dominantly visual content, six to auditory and five can be considered as audiovisual, or bimodal, codes. However, the relative weights of the modalities were not inspected in this study, and thus we cannot state that only visual input affected to the codes labeled here as visual, and vice versa for the auditory labels. Each of the unimodal-labeled codes have most likely been impacted by both modalities to some extent. Figure 10 presents the ordination results from correspondence analysis in Dimensions 1 & 2. The first two dimensions were found to explain 52% (inertia  =  .24) and 20% (inertia  =  .09) of the variance, respectively, while the third dimension accounted for 10% (inertia  =  .05) of the variance, and is not displayed graphically. In addition, the clusters obtained through hierarchical clustering are drawn on top of the ordination.

In Figure 10, stimulus scenes perceived to be similar are displayed close together. Similarly, interview codes attached to similar scenes are displayed close to each other, and close to the corresponding scenes. The Figure reveals what were the most salient attributes that the participants paid attention to when making the categorization decisions for the respective scenes. The five clusters, obtained from hierarchical clustering, are separated in the two-dimensional perceptual map, indicating that the perceptual map is not distorting the underlying high-dimensional similarity space too much. Cluster #1 (*Calm*) resides close to codes reflecting perceived pleasantness and calmness mainly induced by quietness and soundscape with recognizable auditory events. In addition, codes reflecting visual movement are on the opposite side of the map, implying that the visual scene was still, although there were not enough references to codes indicating visual stillness. Cluster #2 (*Still*) is best described in the perceptual map by attributes related to being outdoor or in an open place. The scenes in this category did not have any specific points of interest or events and the overall atmosphere was still. This is reflected in the ordination through the absence of attributes related to temporal aspects, in favor of gist-like attributes of scene openness and background sound.

Cluster #3 (*Noisy*) contained scenes with lots of mechanical noise. The noisiness is well reflected in the perceptual map. In addition, the participants paid attention to the sound source, namely traffic. According to the ordination, Cluster #4 (*Vivid*) is defined to contain scenes with a lot to attend, which is consistent with the clustering result. Especially, attention has been paid to the presence of people in the scenes. Interestingly, a spatial attribute of the soundscape, echo or reverberation, has been frequently mentioned. Finally, Cluster #5 (*Open*) is related with multiple attributes, the most common attribute being *conversation*. Attention was also paid to movement, enclosure and other recognizable sounds. Although all the scenes were somehow open spaces, the attributes related to openness are not ordinated close to the cluster. Apparently, with these scenes, other perceptual properties overruled the scene gist categorization.

### Environmental correspondence of the ordination

**Figure 11:**
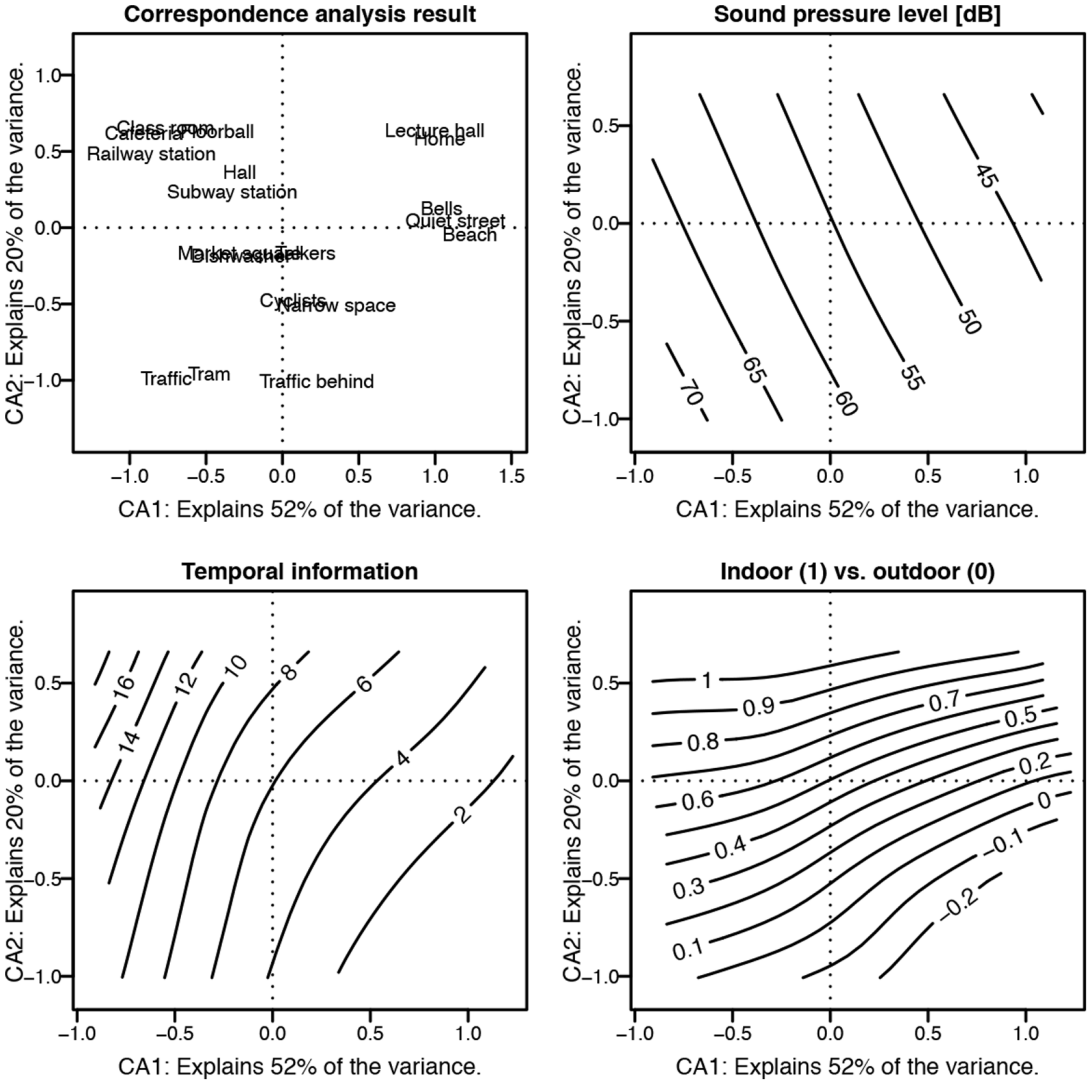
Correspondence analysis for the interview codings with fitted environmental variable surfaces. Top-left panel shows the ordinated scenes, and the next panels display the fitted sound pressure level, indoor vs. outdoor classification, and temporal information surfaces in a clockwise manner.

Objective reasons for the ordination can be searched for from Figure 11, where three environmental variables are computed from the stimulus scenes and fitted to the correspondence analysis (CA) result. The sound pressure level (SPL) was measured at the observation position and it is the A-weighted average SPL over 10 seconds. Temporal information (TI) is defined as the motion difference between adjacent frames. TI is computed by observing the pixel values at the same spatial location at successive frames, as defined in [35]. The maximum standard deviation found in the motion difference was taken to be the TI for a given video sequence. Indoor versus outdoor classification is a binary attribute related to each scene. Value 1 denotes indoor scenes and 0 denotes outdoor. The aim is to fit two-dimensional models to predict the environmental variables from the scenes' CA scores on the two axes.

Sound pressure level, temporal information and indoor vs. outdoor classification are found to have a nearly linear fit to the CA map, the models explaining 87%, 63%, and 82% of the variance in the respective variables. All three fitted surfaces were found statistically significant at *p*  =  .01. Dimension #1 is well described by a combination of increasing SPL and TI levels from right to left. This is in accordance with the codes in Figure 10, where the codes #*Quiet* and #*Calm* can be found on the right and codes #*Movement* and #*Noisy* on the left. Dimension #2 is characterized by the division to indoor scenes on top and outdoor scenes in bottom. This result is verified by the codes #*Outdoor space* and #*Open space* residing in bottom in contrast to #*Large indoor space* and #*Enclosed space* on top. In conclusion, the perceptual map appears to be in accordance with measurable environmental properties, validating the assumptions made in the interview coding process and lending support for the two-dimensional solution for correspondence analysis.

## Discussion

A perceptual map of audiovisual scenes based on a categorization task and a semi-structured interview was derived in our study, confirming our primary hypothesis that a group of people describes natural audiovisual scenes with similar perceptual attributes. The nature of our study was exploratory and thus we cannot precisely identify the relative contributions of the two sensory channels to audiovisual scene perception. From the perceptual map we can draw evidence that the most important perceptual attributes related to the scenes were movement, perceived noisiness, and eventfulness of the scene. Regarding scene gist perception, the gist properties openness and expansion, identified in previous research [49], were found to remain as important factors only in scenes with no salient auditory or visual events. As hypothesized, three environmental variables were found to have a nearly linear fit to the perceptual mapping, adding more support to the assumption that the map is stemming from the physical environment. The perceptual map could have been also predicted by higher-order interpretations such as the social function or the aesthetic interpretation of the depicted spaces, which was not the case here. However, the origins of the attributes were not tested, and different selection of stimulus scenes could have led to different categorization criteria.

Based on the results obtained here, disregarding the higher level function of the space and the meaning of the events taking place at the space, and instead focusing on the physical environment is possible. However, a few scenes were more problematic than the others, for example the scenes #*Class room* and #*Lecture hall* were categorized into the same category by a few participants because they are both known to be related to education, even though the purely physical characteristics of the scenes are strikingly different. Despite the difficulties, the clustering, MDS and CA results show similar overall ordination of the stimulus scenes, which would imply that the underlying perceptual reasoning is prevalent and not resulting from random effects. The experiment assumes categories of environmental scenes to result from perceived similarity. Categorization as a method for studying similarity is justified for perceptually rich stimuli, where information is integrated from multiple sources, and categories can offer useful inferences [50]. However, caution must be taken as the categorization task can influence the perceptual discrimination of the stimuli [51]. Early categorization decisions may affect how people perceive new stimuli and which perceptual dimensions they pay attention to. In this case, all the stimulus scenes were viewed once before the categorization task was introduced, potentially minimizing the task influence. In addition, the participant weights on the perceptual dimensions in MDS analysis (Figure 9) do not show significantly differing weightings for the majority of participants.

An interesting categorization theory has been provided stating that with complex stimuli humans initially do categorization based on simple rules about their properties [52]. Gradually, as the categorization process evolves, learning about discriminating stimuli occurs and the categorization transforms to exemplar-based, and novel stimuli may be rapidly categorized according to the exemplar stimuli without the aid of rules. In this study, the categorization strategy was not under inspection, but informal discussions with the participants after the test revealed, that majority of them had initially used one or two rules, such as loudness and the indoor vs. outdoor classification, to form large categories. Thereafter, with more detailed inspection, the initial categories were divided into smaller ones according to new rules, or, some participants had found exemplar-like scenes, that they used to justify the existence of specific categories. Nevertheless, the task was so complicated that higher-order scene categorization processes cannot be ruled out. The categorization task and the interview may have forced the participants to process the stimuli in an unnatural way that would have eventually affected our results. Further studies are needed to fine-tune the perceptual map possibly by utilizing multiple simpler tasks.

Movement, or the lack thereof, appears to be the most useful attribute in bimodal scene perception. As identified in previous research, visual movement and on-sets quickly draw our attention and direct fixations [4], [53]. The proximity of movement was also reported to be affecting the categorization by some participants, as moving objects nearby were regarded as threatening. The effect of auditory stimulation on the perception of movement requires further studies. The lack of movement is related to perceived calmness and pleasantness, yet, quiet or non-confusing soundscape appears to be needed for the scene to be considered as calm, as demonstrated by the CA (Figure 10). The scene #*Dishwasher* is revelatory in this case, because it has no movement, but very noisy soundscape, and thus it is not perceived as calm. Perceptually calm and pleasant scenes also contained visual elements present in the nature (such as trees, grass and the sea), which is in accordance with previous research [31], although naturalness was not frequently mentioned by the participants and thus not present in the perceptual map. The absence of nature-related attributes may result from the fact that all the stimulus scenes were recorded in dominantly man-made urban environments.

Even though the stimulus duration was 15 s, scene gist dimensions openness and expansion seem to be robust features also in dynamic audiovisual perception. These dimensions are perceived within a single glance of the visual world [13], but still they were meaningful to humans despite the long stimulus duration and the complex categorization task. There were four interview codes related to the perceived size of the space or indoor vs. outdoor classification of the scene. Especially, when the scene had no salient events, as was the case with #*Narrow space* and #*Cyclists*, the gist properties were the things that the participants noticed. Related to the size of a space, the perception of reverberation or echo was frequently mentioned with large indoor spaces. In previous research on soundscape perception, the spatial factors were seldom mentioned by test participants [21]. Here, potentially, the accompanying visual stimulus guided also the auditory perception towards the acoustic properties of the space, because large indoor spaces are known to be reverberant. However, this phenomenon may also be related to different listening strategies: In a real environment people are prone to apply *everyday listening*, while in a laboratory the listening strategy is more analytic, *musical listening* [54]. Moreover, in a laboratory setting the participants are found to be more affected by the spatial attributes of the sound, while in a real environment the sound sources are described more precisely [55].

The auditory scene attributes appear to be divided to quiet scenes, scenes with recognizable or meaningful sound events (conversation), background sound and noise. The division is in accordance with the finding of event sequences and amorphous sequences [20]. In addition, recognized sounds vs. noise separation reflecting the perceived valence is evident in Figure 10, as found previously in soundscape studies [22], [23] indicating that the emotional state induced by the soundscape is meaningful to humans even with complex audiovisual stimuli. Evidence for auditory gist perception [56], apart from auditory streaming, cannot be directly drawn based on this study, but the notion of amorphous sequences, or background sound, that was perceived but not analyzed further, would suggest that some kind of an overall representation of the soundscape is obtained, while attention is directed towards more salient events.

Based on our exploratory results, humans tend to focus on perceived movement, noisiness and eventfulness when describing their experience of real-life urban environments. However, the relative weights of the modalities cannot be quantified with our data, and future studies will be needed. Moreover, some of the elicited attributes, namely #*Calm* and #*Pleasant*, may not be readily usable for modeling, but need more elaborate investigation to relate them to perceivable scene properties. The effect of top-down information on real-world scene perception through attention allocation is also worth investigating. Arousal can have modulating effects on selective attention giving more attentional resources for the processing of the most salient stimuli while decreasing the processing of low priority stimuli [57]. Previous studies on overt attention have concentrated on simple stimuli and tasks, for example by asking the participants to focus on a certain spatial location. In the real-world, however, the task might be not to get run over by a car that you can hear but not yet see, which is a lot more arousing experience, and probably affects the scene processing priorities. Therefore, we would like to draw attention towards the concept of audiovisual gist that builds on the visual gist perception and is biased by the auditory scene, to better reflect the situation in the real world. We hypothesize that audiovisual processing of the scene gist results in dissimilar perception of the reality in further processing, when compared to unimodal gist processing. For example, the perceived calmness of a scene could rapidly arise from the combination of a visual gist interpretation and a cursory overview of the soundscape. Thereafter, the acquired audiovisual gist could guide the more detailed visual and auditory exploration, and probably be related to the state of arousal experienced by the perceiver, and eventually affect how we classify scenes. However, our study did not directly provide information about gist effects due to the length of the stimulation, and further studies are needed to link the effect of scene gist to the results presented here. Similarly, we can only hypothesize that the elicited perceptual attributes are stemming from the perceptually salient aspects of the scenes, as saliency as a phenomenon was not directly studied here. Further studies are needed to test the attributes' relation to audiovisual saliency.

## Conclusions

In conclusion, audiovisual perception of natural scenes was studied in this work. Perceptual similarity of 19 natural scenes reproduced with an immersive audiovisual display was evaluated through a categorization task followed by a semi-structured interview. Our exploratory results point out that a two-dimensional perceptual map of a set of natural audiovisual scenes depicting real-life urban environments is obtainable, and movement, noisiness and eventfulness of the scene are the most important perceptual attributes. Scene gist properties describing the size and openness of the space were present in the results, when there was an absence of salient events. However, we cannot state what exactly were the individual contributions of the modalities, or that the elicited perceptual attributes are stemming from perceptual saliency.

Overall, when considering a real-world scene, including the auditory modality has an enormous impact on our perception. We propose that the study of natural scene perception should move forward to better understand the processes behind real-world scene processing including multiple modalities. Moreover, the question of how do we arrive from the gist of the visual scene to a thorough understanding of the multimodal reality is worth investigating. The current work is an exploratory study that demonstrates the existence of frequently used perceptual scene attributes. Further studies are needed to quantify the individual contributions of auditory and visual modalities on audiovisual perception of natural scenes.
